# Prey Preference and Life Table of *Amblyseius orientalis* on *Bemisia tabaci* and *Tetranychus cinnabarinus*

**DOI:** 10.1371/journal.pone.0138820

**Published:** 2015-10-05

**Authors:** Xiaoxiao Zhang, Jiale Lv, Yue Hu, Boming Wang, Xi Chen, Xuenong Xu, Endong Wang

**Affiliations:** 1 Lab of Predatory Mites, Institute of Plant Protection, Chinese Academy of Agricultural Sciences, Beijing, China; 2 Syngenta Biotechnology (China) Co., Ltd., Beijing, China; French National Institute for Agricultural Research (INRA), FRANCE

## Abstract

*Amblyseius orientalis* (Ehara) (Acari: Phytoseiidae) is a native predatory mite species in China. It used to be considered as a specialist predator of spider mites. However, recent studies show it also preys on other small arthropod pests, such as *Bemisia tabaci* (Gennadius) (Hemiptera: Aleyrodidae). Experiments were conducted to investigate (1) prey preference of *A*. *orientalis* between *Tetranychus cinnabarinus* (Boisd.) (Acari: Tetranychidae) and *B*. *tabaci*, and (2) development, consumption and life table parameters of *A*. *orientalis* when reared on *T*. *cinnabarinus*, *B*. *tabaci* or a mix of both prey species. When preying on different stages of *T*. *cinnabarinus*, *A*. *orientalis* preferred protonymphs, whereas when preying on different stages of *B*. *tabaci*, *A*. *orientalis* preferred eggs. When these two most preferred stages were provided together (*T*. *cinnabarinus* protonymphs and *B*. *tabaci* eggs), *A*. *orientalis* randomly selected its prey. *Amblyseius orientalis* was able to complete its life cycle on *B*. *tabaci* eggs, *T*. *cinnabarinus* protonymphs, or a mix of both prey. However, its developmental duration was 53.9% and 30.0% longer when reared on *B*. *tabaci* eggs than on *T*. *cinnabarinus* and a mix of both prey, respectively. In addition, it produced only a few eggs and its intrinsic rate of increase was negative when reared on *B*. *tabaci* eggs, which indicates that *B*. *tabaci* is not sufficient to maintain *A*. *orientalis* population. The intrinsic rates of increase were 0.16 and 0.23 when *A*. *orientalis* was fed on the prey mix and *T*. *cinnabarinus*, respectively. These results suggest that although *B*. *tabaci* is a poor food resource for *A*. *orientalis* in comparison to *T*. *cinnabarinus*, *A*. *orientalis* is able to sustain its population on a mix of both prey. This predatory mite may thus be a potential biological control agent of *B*. *tabaci* when this pest co-occurs with the alternative minor pest *T*. *cinnabarinus*.

## Introduction

*Bemisia tabaci* (Gennadius) (Hemiptera: Aleyrodidae) is one of the most serious agricultural pests that injure various crops, vegetables, and ornamental plants not only in China, but also worldwide [1–2]. Whiteflies cause great yield loss and economic injury not only by injuring host plants directly through leaf piercing, sap sucking and secreting honeydew, but also transmitting numerous plant viruses [3–6]. In China, *B*. *tabaci* generally causes ca. 15% yield loss, and up to 75% yield loss when it occurs at severe densities [7].

Efforts are being developed to effectively control *B*. *tabaci*. Chemical control induces environmental and food safety problems [8–10], and biological control has been an important alternative for a long time [11–12]. Some predatory mites are effective predators of whiteflies, such as *Amblyseius swirskii* (Athias-Henriot) (Acari: Phytoseiidae), which has been widely used in Europe and North America [13–15]. However, this species is exotic to Asia. Preliminary studies indicated that *A*. *swirskii* might have negative impacts on native phytoseiid mite populations if introduced [16–17]. In addition, biological control is sometimes considered as ‘unreliable’ because many natural enemies are environmentally sensitive and their performances vary under different conditions [12]. Therefore, it is always valuable to develop new biological control agents to provide more choices. Currently, limited data about whitefly control capability of native predatory mite species are available in China.

*Amblyseius orientalis* (Ehara) (Acari: Phytoseiidae) is a native predatory mite species widely distributed in China. It used to be considered as a specialist predator of spider mites [18], and has been applied in orchards as a biological control agent of spider mites since the 1980s. Early studies showed that *A*. *orientalis* reduced *Panonychus citri* density by 68.86% five days after released in orange orchards [19], and led to up to 93.4% control of *Panonychus ulmi* and *Tetranychus viennensis* when released in apple orchards [20]. Recent studies showed that this species is actually a generalist predator. It is able to be mass reared with *Carpoglyphus lactis*, a storage pest mite that is often used as an alternative prey in mass production of predatory mites [Patent: CN 201110456703], and it also preys on thrips and whiteflies [21]. It is valuable to estimate the potential of *A*. *orientalis* as a generalist predator of other pests, such as whiteflies [1].

Previous studies show that *A*. *orientalis* was able to complete its life cycle on *B*. *tabaci*, but its oviposition rate was ca. 90% lower than those when reared on spider mites [21–22]. For generalist predators, a mixed diet including multiple prey species might not only lead to better control efficiency of each pest species, but also lead to higher chances for population establishment and greater population increase than using each prey species separately [23–26]. Under natural circumstances, whiteflies often occur together with *Tetranychus* spp., such as *Tetranychus cinnabarinus* (Boisd.) (Acari: Tetranychidae) [1, 25]. Therefore, it is valuable to test whether *A*. *orientalis* is able to control the major pest *B*. *tabaci* co-occurring with the minor pest *T*. *cinnabarinus*. To investigate predation and population dynamics of *A*. *orientalis* when both these two prey species are available, two experiments were conducted in the present study: (1) prey preference of *A*. *orientalis* in three different treatments: between two stages of *B*. *tabaci*, among three stages of *T*. *cinnabarinus*, or between the most preferred stages of each prey species, and (2) the impact of three prey treatments (*B*. *tabaci* only, *T*. *cinnabarinus* only, and a mix of both prey species) on *A*. *orientalis* development, reproduction, and population success.

## Materials and Methods

### Mites and whiteflies colonies

*Amblyseius orientalis* was obtained from a colony maintained in the Lab of Predatory Mites, Institute of Plant Protection, Chinese Academy of Agricultural Sciences. The colony was established with individuals collected from soybean fields of Changli Research Institution of Pomology, Hebei Academy of Agricultural and Forestry Sciences (119°09’E, 39°43’N), with the permission from Prof. Lichen Yu, and has been maintained on *C*. *lactis*, in a climate chamber at 25±1°C, 80%±5% RH and 16L: 8D for multiple years. The *C*. *lactis* colony was obtained from Prof. Dr. Qinghai Fan, Fujian University of Agricultural and Forestry Sciences. *Bemisia tabaci* was obtained from Assistant Prof. Yubo Wang, Dryland Farming Research Institute, Hebei Academy of Agricultural and Forestry Sciences, and was reared on 2-week-old bean (*Phaseolus vulgaris*) seedlings in climate boxes (22±1°C, 60%±5% RH and 16L: 8D). *Tetranychus cinnabarinus* was collected from strawberry fields of Changping Agricultural Technology Extension Station, Beijing (116°12’E, 40°13’N), with the permission from station director Baowen Liu, and was reared on 2-week-old bean seedlings at the same conditions as for rearing *A*. *orientalis*. Bean seedlings used for colony maintenance were reared at 25±1°C, 70%±5% RH and 16L: 8D.

### Experimental unit

**Fig 1:**
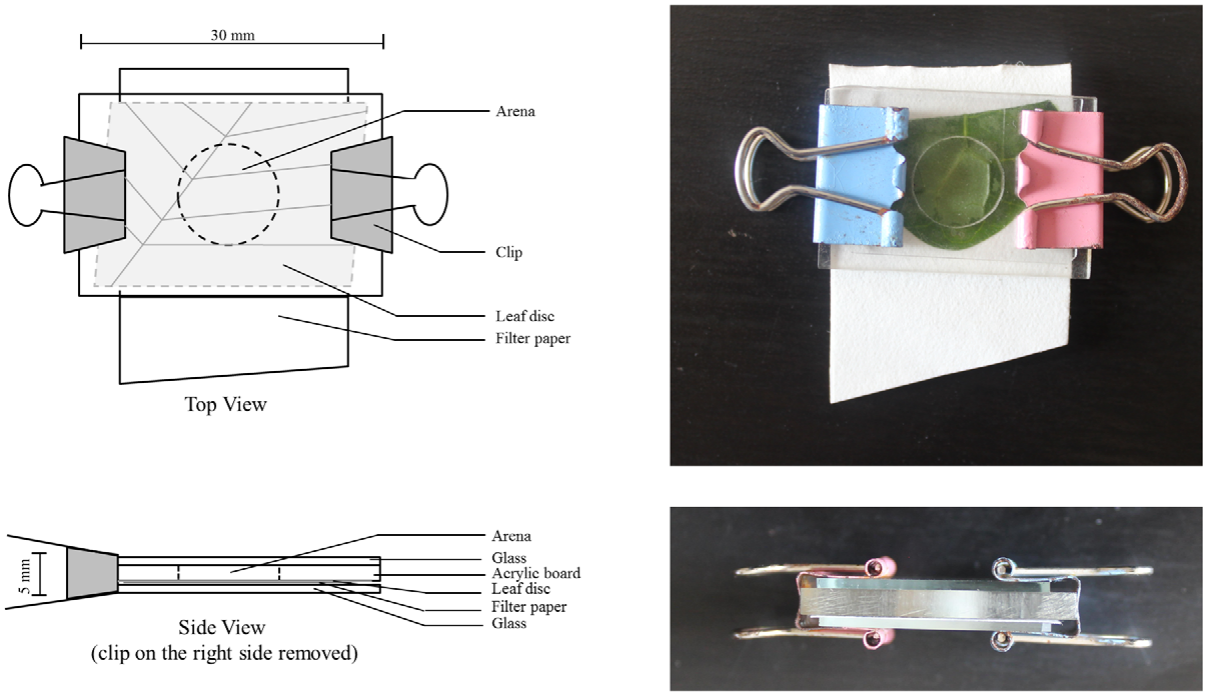
Schematic views and photographs of the experimental unit.

In both preference and life table experiments (experiments (1) and (2) respectively), *A*. *orientalis* was reared individually in 10 (dia.)×3 (h.) mm^3^ arena, which was built with a transparent acrylic board (30×20×3mm^3^) with a 10mm diameter hole in the center, sealed on one side with a piece of bean leaf disc (made of first leaves of 2-week-old bean seedlings as used for colony maintenance), a piece of filter paper, and a piece of rectangular glass (30×20×1mm^3^), and on the other side with another piece of rectangular glass (30×20×1mm^3^). The 5 layers were tightly clipped together on both ends to avoid mite escaping (Fig 1). Preliminary observations show that leaves keep fresh for ca. 2 days in our experimental unit. Experimental units were placed in climatic chambers at 25±1°C, 70%±5% RH and 16L: 8D.

### Prey preference between *B*. *tabaci* and *T*. *cinnabarinus*

In this experiment, the predator *A*. *orientalis* was exposed to three distinct treatments to estimate its prey preference. The first two treatments estimated preference of *A*. *orientalis* between two *B*. *tabaci* stages (eggs or 1^st^ instar larvae) and among three *T*. *cinnabarinus* stages (eggs, larvae, or protonymphs), respectively. A preliminary study with each prey species and stage provided in isolation suggest that later stages of these species are less preferred by *A*. *orientalis* and were therefore not tested in the present study [21]. In each treatment, 20 experimental units were prepared. In each experimental unit, 10 individuals of each stage were provided to a female *A*. *orientalis* adult. All females were starved for 24 hours before being used in preference experiments to standardize hunger levels. The number of prey consumed within 12 hours was recorded. Since prey were not able to escape from the experimental unit, all missing prey were considered consumed by the predator.

The 3^rd^ treatment was conducted to estimate *A*. *orientalis* preferences between the most preferred stages of each of the two prey species (*B*. *tabaci* eggs and *T*. *cinnabarinus* protonymphs, as obtained from the previous experiments). Three sets of prey density combinations were provided to *A*. *orientalis* adult females starved for 24 hours: (1) balanced densities with 10 individuals of each prey species, (2) 10 *B*. *tabaci* eggs and different numbers (15, 20, 25, 30, 35) of *T*. *cinnabarinus* protonymphs, and (3) 10 *T*. *cinnabarinus* protonymphs and different numbers (15, 20, 25, 30, 35) of *B*. *tabaci* eggs. A minimum of 15 replicates were prepared for each density. The number of each prey species consumed within 12 hours was recorded.

In each preference experiment, the prey preference index (α) was estimated using (Eq 1), modified from Chesson’s preference index [27], where *N*_*i*_ is the number of the *i* ^th^ prey type available, and *N*_*ai*_ is the number of the *i* ^th^ prey type consumed. For each individual test, the preference index of the most preferred food type equals 1.

$$\alpha \;=\;\left(N_{ai}/N_{i}\right)\;/Max\left(\frac{N_{ai}}{N_{i}}\right)$$ (1)

Mean prey preference indices to each prey type were compared with paired t-test and one-way ANOVAs when there were 2 or 3 choices, respectively. When ANOVA was conducted, multiple comparisons were also conducted with Tukey HSD test. All mean comparisons with p<0.05 were considered to have statistical significant differences. In the 3^rd^ experiment, linear regressions were conducted to estimate the impact of the proportion of *T*. *cinnabarinus* in the prey mix on the number of both prey species consumed by *A*. *orientalis*. All analyses were processed with SPSS 19.0.

### Impact of prey type on population biology and life-table parameters of *A*. *orientalis*

A second experiment was performed to estimate *A*. *orientalis* life table parameters when preying on *T*. *cinnabarinus* only, *B*. *tabaci* only, and a mix of both prey, respectively. The experiment was started with 100, 65, and 80 synchronic eggs (laid within 6 hours, considered as 3 hours old on average when experiment started) for *T*. *cinnabarinus*, *B*. *tabaci* and the prey mix treatments, respectively. Fewer numbers of eggs were used for the *B*. *tabaci* and the prey mix treatments due to limited availability of *B*. *tabaci* eggs. After each *A*. *orientalis* larval emergence, 35 *T*. *cinnabarinus* protonymphs, 25 *B*. *tabaci* eggs, or a combination of 15 *T*. *cinnabarinus* protonymphs and 25 *B*. *tabaci* eggs were provided daily, for each of the three treatments, respectively. A smaller number of *T*. *cinnabarinus* protonymphs were provided in the prey mix treatment to match our objective to evaluate the performance of *A*. *orientalis* when *B*. *tabaci* is the major pest and *T*. *cinnabarinus* is a secondary pest. The amount of prey exceeded maximum daily consumption rate of the predator based on preliminary observations.

Survival and stage of each *A*. *orientalis* individual was recorded every 12 hours during the immature stages. For individuals that entered the next stage between two observations, the molting timing was taken as the midpoint time between the two observations. Newly emerged adult females were paired with males for 24 hrs. Preliminary observations show that *A*. *orientalis* adults mate within two hours after being paired and mating usually lasts for two hours, similar to previous reports of other Phytoseiid mite species [28]. Daily consumption rate and fecundity of each female were recorded until it dies. Developmental and fecundity data were right-skewed, and were rank-transformed for further analyses [29–31].

The conversion rate from daily biomass intake (*I*) to the daily fecundity (*F*) is termed as the prey-offspring conversion rate (*γ*), and was estimated using (Eq 2), modified by Hayes (1988) from Beddington et al. (1976) [32–33], where *M* indicates prey consumed to maintain basal metabolism. Empirical estimates of *γ* and *M* were obtained through linear regressions of daily prey consumption against daily fecundity.

F = γ×(I−M) (2)

Life tables were constructed from the observed age-specific survival rate (*l*_*x*_) and age specific fecundity rate (*m*_*x*_) of *A*. *orientalis* females. The following life table parameters were estimated: the intrinsic rate of population increase (*r*_*m*_), the finite rate of population increase (*t*), the net reproductive rate (R_0_), the mean generation time (*T*), and the doubling time (*t*) using Eqs (3a–3e) [34–35].

$$\sum_{x=0}^{\infty }{\text{e}}^{-{\text{r}}_{\text{m}}\left(\text{x}+1\right)}{\text{l}}_{\text{x}}{\text{m}}_{\text{x}}\;=\;1$$ (3a)

$$\lambda \;=\;e^{r_{m}}$$ (3b)

$$R_{0}\;=\;\sum_{x=0}^{\infty }l_{x}m_{x}$$ (3c)

$$T\;=\;lnR_{0}/r_{m}$$ (3d)

$$t\;=\;ln2/r_{m}$$ (3e)

Variances of *M*, *γ*, and life table parameters were estimated using the bootstrap technique with 1000 repeated samples [36–37]. Bootstrap resamplings were conducted with R version 3.2.0. [38]. One-way ANOVAs were conducted to compare mean developmental durations (rank-transformed data), fecundity (rank-transformed data), and life table parameters of *A*. *orientalis* in the three treatments. Two-way ANOVA was conducted to estimate the impact of both prey type and predator growth stage on *A*. *orientalis* consumption rate. Multiple comparisons were also conducted with Tukey HSD test. All mean comparisons with p<0.05 were considered to have statistical significant differences. Mean comparison analyses were processed with SPSS 19.0.

## Results

### Prey preference between *B*. *tabaci* and *T*. *cinnabarinus*

*Amblyseius orientalis* significantly preferred *B*. *tabaci* eggs to *B*. *tabaci* 1^st^ instar larvae (t = 6.560; df = 19; p<0.001) (Table 1). No significant difference in predation among the three *T*. *cinnabarinus* stages was detected (F = 0.892; df = 2, 57; p = 0.416), although a slightly higher consumption of protonymphs than eggs and larvae was observed (Table 2).

**Table 1:** Prey preference of *A*. *orientalis* between *B*. *tabaci* eggs and 1st instar larval.

Prey Stages	Initial Density	n	Number Consumed	Preference Index α
**Eggs**	10	20	5.80±0.46a	0.97±0.02a
**1** ^**st**^ **Instar Larvae**	10	20	2.05±0.33b	0.40±0.07b

Means ± SE followed by different lowercase letters in the same column were significantly different at p = 0.05.

**Table 2:** Prey preference of *A*. *orientalis* among *T*. *cinnabarinus* eggs, larvae and protonymphs.

Prey Stages	Initial Density	n	Number Consumed	Preference Index α
**eggs**	10	20	3.25±0.23a	0.81±0.04a
**larvae**	10	20	3.10±0.23a	0.76±0.05a
**protonymphs**	10	20	3.48±0.24a	0.85±0.05a

Means ± SE followed by different lowercase letters in the same column were significantly different at p = 0.05.

**Fig 2:**
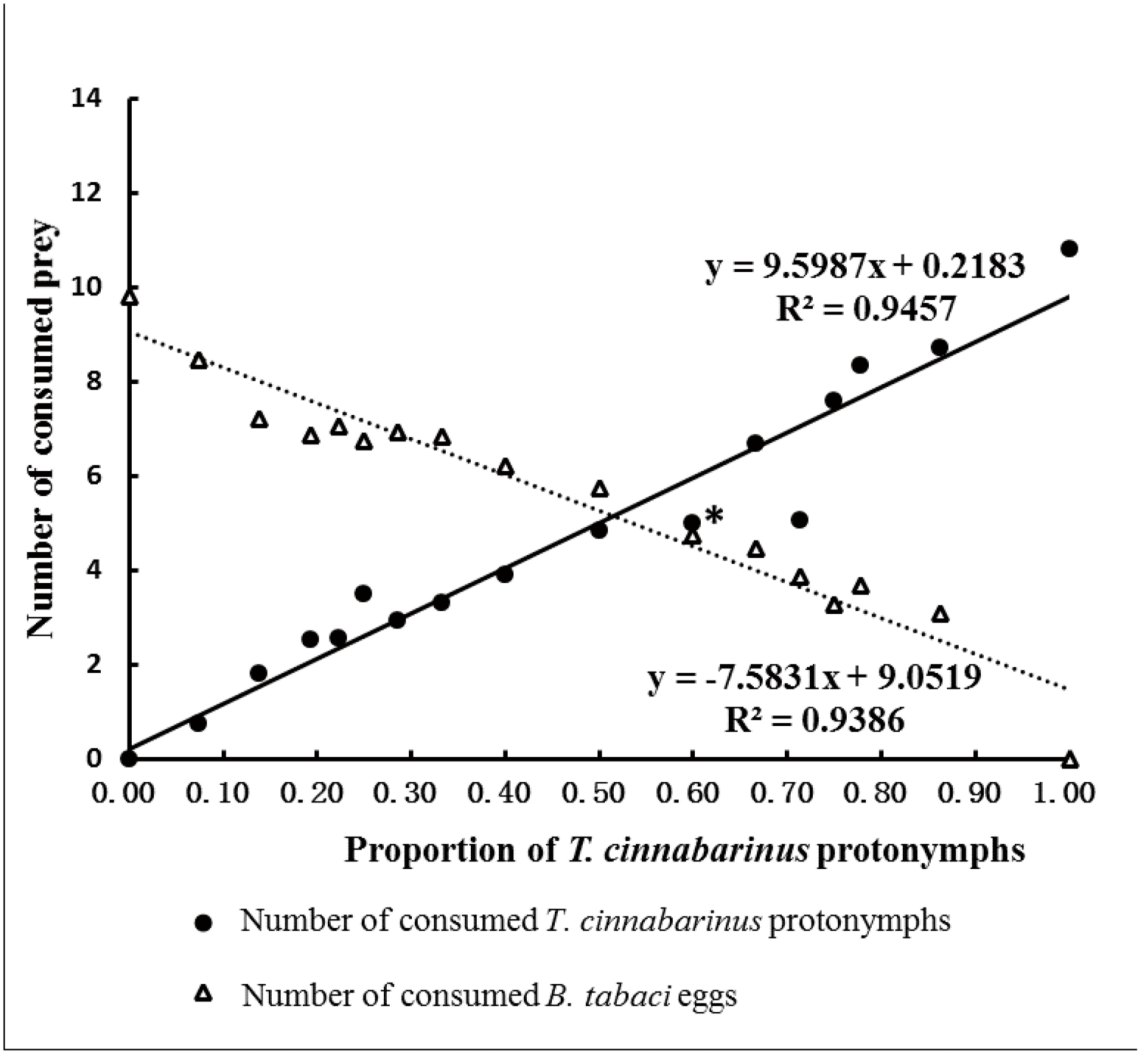
Impact of the proportion of *T*. *cinnabarinus* and *B*. *tabaci* in the mixed diet on prey consumption of *A*. *orientalis*. (* refers to the proportion of *T*. *cinnabarinus* protonymphs where significant preference for whitefly eggs was observed).

When both prey species were provided together, the overall preference index of *A*. *orientalis* to *T*. *cinnabarinus* protonymphs did not differ significantly from that of *A*. *orientalis* to *B*. *tabaci* eggs (t = -1.940, df = 201, p = 0.054) (Table 3). Among all density combinations, a significant preference to *B*. *tabaci* eggs was observed when 10 *B*. *tabaci* eggs and 15 *T*. *cinnabarinus* protonymphs were provided (t = -2.934; df = 14; p = 0.011) (Table 3, Fig 2). The number of each prey type consumed matched their available proportion (Fig 2). The results suggest that *A*. *orientalis* randomly preys *B*. *tabaci* eggs and *T*. *cinnabarinus* protonymphs.

**Table 3:** Prey preference of *A*. *orientalis* between *B*. *tabaci* eggs and *T*. *cinnabarinus* protonymphs.

Initial Density			Number of Prey Consumed			Preference Index α		Paired T-test of α		
*B*. *tabaci* Eggs	*T*. *cinnabarinus* Protonymphs	n	Total	*B*. *tabaci* Eggs	*T*. *cinnabarinus* Protonymphs	*B*. *tabaci* Eggs	*T*. *cinnabarinus* Protonymphs	t	df	p
**35**	**10**	20	9.60±0.80	7.05±0.64	2.55±0.39	0.72±0.07A	0.78±0.07A	0.451	19	0.657
**30**	**10**	16	10.25±0.63	6.75±0.72	3.50±0.43	0.66±0.08A	0.89±0.07A	1.858	15	0.083
**25**	**10**	15	9.87±0.75	6.93±0.76	2.93±0.37	0.79±0.08A	0.79±0.08A	0.049	14	0.961
**20**	**10**	27	10.15±0.84	6.85±0.74	3.30±0.37	0.81±0.06A	0.77±0.05A	-0.379	26	0.707
**15**	**10**	23	10.13±0.52	6.22±0.53	3.91±0.47	0.82±0.06A	0.72±0.06A	-0.850	22	0.404
**10**	**10**	26	10.58±0.58	5.73±0.36	4.85±0.42	0.89±0.04A	0.75±0.05A	-1.821	25	0.081
**10**	**15**	15	9.73±0.77	4.73±0.43	5.00±0.57	0.94±0.04B	0.67±0.07A	-2.934	14	0.011
**10**	**20**	15	11.13±0.97	4.47±0.68	6.67±0.88	0.85±0.06A	0.72±0.08A	-1.023	14	0.323
**10**	**25**	15	8.93±0.68	3.87±0.68	5.07±0.45	0.83±0.09A	0.60±0.08A	-1.459	14	0.167
**10**	**30**	15	10.87±1.48	3.27±0.45	7.60±1.28	0.79±0.09A	0.69±0.07A	-0.628	14	0.540
**10**	**35**	15	12.00±1.19	3.67±0.61	8.33±1.02	0.78±0.09A	0.68±0.08A	-0.673	14	0.512
**Total**		-	-	-	-	0.81±0.02A	0.74±0.02A	-1.940	201	0.054

Means ± SE followed by different uppercase letters in the same row were significantly different at p = 0.05.

### Impact of prey type on population biology and life-table parameters of *A*. *orientalis*

Among all eggs used in this experiment, 93, 76, and 57 developed to adults when reared on *T*. *cinnabarinus* protonymphs, the prey mix and *B*. *tabaci* eggs, respectively, among which, 43, 44 and 32 were females. Biological characteristics and life table parameters were estimated based on data obtained from female individuals. *Amblyseius orientalis* was able to complete its life cycle on all the three treatments. Developmental duration of eggs did not differ significantly among the three treatments, while for other immature stages and the pre-oviposition stage, the shortest developmental duration was observed when *T*. *cinnabarinus* protonymphs were provided, followed by the prey mix and *B*. *tabaci* eggs treatments (Table 4).

**Table 4:** Impact of prey type on duration (days) of life stages and reproduction of *A*. *orientalis*.

Biological Characteristics		Treatments			ANOVA Analyses		
		*T*. *cinnabarinus* Protonymphs (n = 43)	Prey Mix (n = 44)	*B*. *tabaci* Eggs (n = 32)	F	df	*p*
**Developmental Durations and Longevity (days)**							
	**Egg**	1.95±0.02a	1.99±0.03a	2.04±0.04a	1.738	2,116	0.180
	**Larval**	0.70±0.02a	0.95±0.03b	1.31±0.03c	134.859	2,116	<0.001
	**Protonymph**	0.98±0.01a	1.18±0.04b	2.02±0.06c	193.701	2,116	<0.001
	**Deutonymph**	0.99±0.02a	1.32±0.07b	1.73±0.04c	66.31	2,116	<0.001
	**Egg-Adult**	4.62±0.03a	5.43±0.11b	7.10±0.10c	204.273	2,116	<0.001
	**Pre-Oviposition**	1.14±0.06a	2.35±0.17b	3.27±0.26c	66.245	2,105	<0.001
	**Oviposition**	20.93±0.76b	26.82±1.01c	2.81±0.46a	317.328	2,105	<0.001
	**Post-Oviposition**	70.53±3.09c	23.90±2.14b	11.67±1.49a	45.083	2,105	<0.001
	**Female Longevity**	97.21±3.00c	58.49±2.42b	26.54±1.20a	171.790	2,116	<0.001
**Fecundity**							
	**Daily Fecundity**	1.88±0.03c	1.28±0.04b	0.65±0.09a	83.039	2,116	<0.001
	**Total Fecundity**	39.49±1.57b	34.66±1.72b	1.69±0.33a	411.881	2,116	<0.001
	**Proportion of Females**	0.63±0.00b	0.62±0.01b	0.45±0.07a	16.906	2,105	<0.001

Means ± SE within a row followed by different lowercase letters are significantly different at p = 0.05.

*Amblyseius orientalis* that used *B*. *tabaci* eggs as prey had the shortest oviposition duration and longevity, and the lowest daily fecundity and cumulative fecundity. Among the two other treatments, the oviposition duration of *A*. *orientalis* on the prey mix was 28% longer than that on *T*. *cinnabarinus* protonymphs. The post-oviposition duration and female longevity were 66% and 40% shorter, respectively, when reared on the prey mix than on *T*. *cinnabarinus* protonymphs (Table 4). Daily fecundity of *A*. *orientalis* on *T*. *cinnabarinus* protonymphs was 47% higher than that on the prey mix, while the cumulative fecundity of these two treatments does not differ significantly (Table 4). No significant difference was observed between the proportions of *A*. *orientalis* female offspring when reared on *T*. *cinnabarinus* protonymphs and on the prey mix, but both were higher than that on *B*. *tabaci* eggs (Table 4).

**Fig 3:**
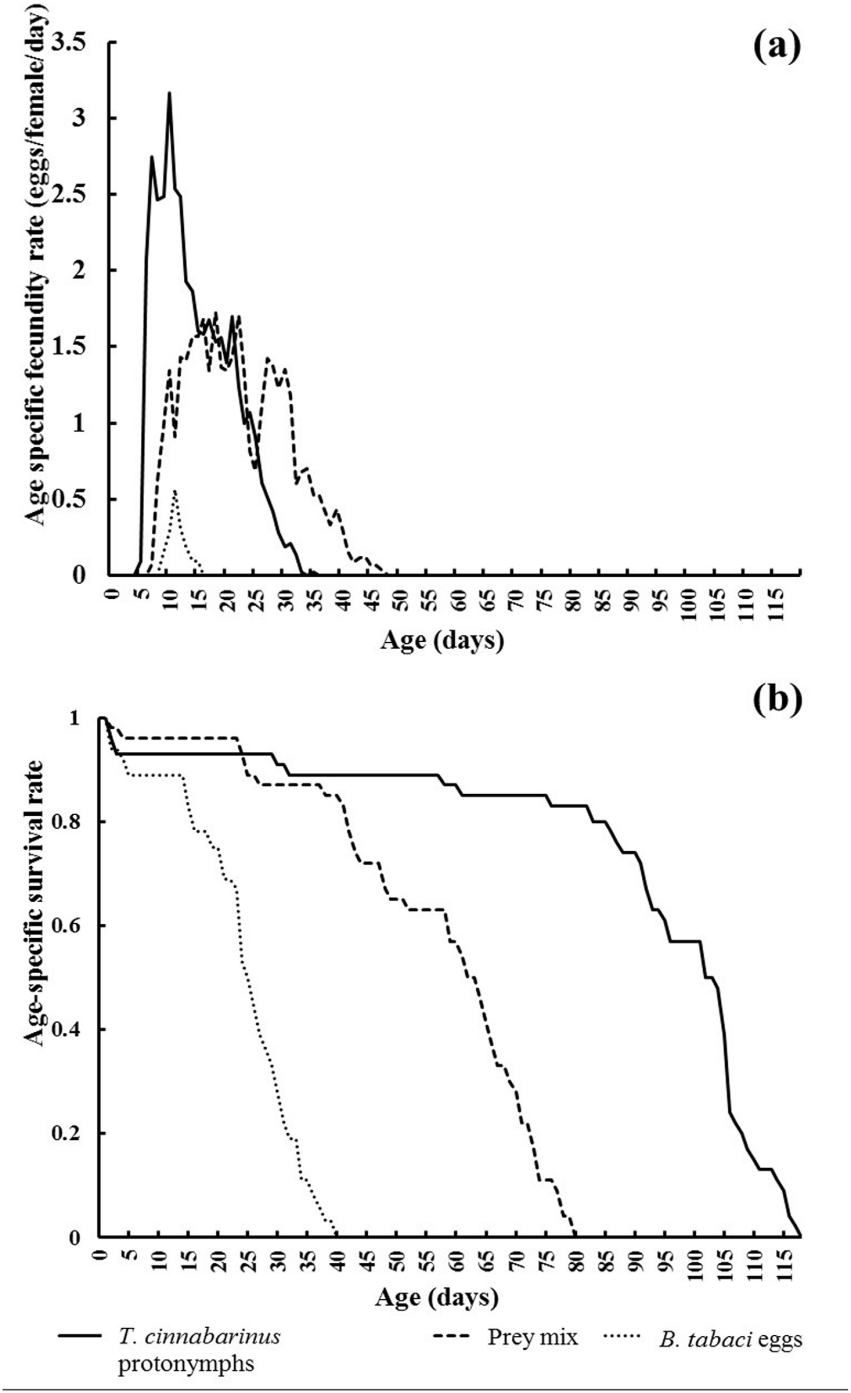
Age-specific survival (a) and fecundity (b) of *A*. *orientalis* when provided with *T*. *cinnabarinus* only, *B*. *tabaci* only, or a mixed diet.

Fig 3(a) summarizes *A*. *orientalis* female age-specific survival rate (*l*_*x*_), while Fig 3(b) summarizes its age-specific fecundity curves (*m*_*x*_) for the 3 treatments. The peak daily fecundity occurred 7, 10 and 3 days after adult emergence of *A*. *orientalis* reared on *T*. *cinnabarinus* protonymphs, the prey mix, and *B*. *tabaci* eggs, respectively.

The total prey consumption of *A*. *orientalis* was significantly affected by both prey type (F = 169.208; df = 2, 659; p<0.001), predator growth stage (F = 437.264; df = 5, 659; p<0.001), and the 2 way interaction (F = 62.374; df = 10, 659; p<0.001). For each of the three treatments, daily consumption rate of *A*. *orientalis* was higher at oviposition duration and pre-oviposition durations than at other stages (Table 5). When *A*. *orientalis* fed on *T*. *cinnabarinus*, its daily consumption rates at oviposition and pre-oviposition durations were ca. twice as high as that in the other two treatments, while much smaller differences were observed for other stages across the three treatments (Table 5).

**Table 5:** Impact of prey type on daily consumption of *A*. *orientalis*.

	Daily Consumption		
	(Min.~Max.)		
Life Stages	*B*. *tabaci* Eggs	*T*. *cinnabarinus* Protonymphs	Prey Mix
**Larva Stage**	1.02±0.18aA	1.07±0.19aA	0.98±0.16aA
	(0~3)	(0~4)	(0~4)
**Protonymph Stage**	4.89±0.38bB	3.00±0.36bA	4.11±0.23cB
	(1~15)	(0~8)	(1~8)
**Deutonymph Stage**	7.14±0.62cA	7.56±0.35dA	6.43±0.35dA
	(0~17)	(4~18)	(1~12)
**Pre-Oviposition Stage**	8.63±0.32cA	14.54±0.42eB	8.09±0.37eA
	(2~20)	(5~30)	(0~23)
**Oviposition Stage**	8.05±0.80cA	19.59±0.38fC	9.70±0.24fB
	(1~22)	(4~34)	(0~25)
**Post-Oviposition Stage**	4.72±0.30bB	5.37±0.20cB	2.91±0.19bA
	(1~14)	(0~13)	(0~16)
**Immature Stages Mean**	6.12±0.21A	8.55±0.11B	6.67±0.11A
**Adult Stages Mean**	4.71±0.30A	3.88±0.30A	3.84±0.25A

Means ± SE within a column followed by different lowercase letters are significantly different at p = 0.05. Means ± SE within a row followed by different uppercase letters are significantly different at p = 0.05.

Table 6 summarizes estimated daily metabolism and prey-offspring conversion rate of *A*. *orientalis* in the three treatments. The estimated amount of prey consumed for basal metabolism was higher when *T*. *cinnabarinus* protonymphs were provided than when *B*. *tabaci* eggs or the prey mix were provided. When the prey mix were provided, *A*. *orientalis* showed higher conversion rate of each prey species (0.15 for *B*. *tabaci*, 0.16 for *T*. *cinnabarinus*) than that when each prey species was provided separately. The lowest conversion rate (0.02) was observed when *B*. *tabaci* eggs were provided.

**Table 6:** Estimated daily biomass intake allocation of *A*. *orientalis* as affected by prey species.

		Prey-Offspring Conversion Rate			
Prey Treatments	Basal Metabolism	*B*. *tabaci* Eggs	p_1_	*T*. *cinnabarinus* Protonymphs	p_2_
***B*. *tabaci* Eggs**	1.81±0.06	0.02±0.00	0.003	-	-
***T*. *cinnabarinus* Protonymphs**	4.60±0.01	-	-	0.11±0.00	<0.001
**Prey Mix**	2.33±0.01	0.15±0.00	0.000	0.16±0.00	<0.001

Significant effects of prey types on all the life table parameters were observed (Table 7). When *B*. *tabaci* was used as the prey, both intrinsic rate of increase and doubling time of *A*. *orientalis* were negative (Table 7). Among the other two treatments, faster population increase was expected when *T*. *cinnabarinus* protonymphs was used as the prey, but *A*. *orientalis* population also increased on the prey mix.

**Table 7:** Impact of prey type on life table parameters of *A*. *orientalis*.

	Prey			Parameters of Significance		
Parameter	*T*. *cinnabarinus* Protonymphs	Prey Mix	*B*. *tabaci* Eggs	F	df	*p*
**Intrinsic Rate of Increase, r**_**m**_ **(day** ^**−1**^ **)**	0.23±0.00c	0.16±0.00b	-0.03±0.00a	191051.318	2,2997	<0.001
**Finite Rate of Population Increase, λ**	1.26±0.00c	1.17±0.00b	0.97±0.00a	234985.496	2,2997	<0.001
**Net Reproduction Rate, Ro**	23.36±0.04c	20.57±0.04b	0.67±0.00a	153398.577	2,2997	<0.001
**Mean Generation Time,T (days)**	13.46±0.01b	19.27±0.01c	13.31±0.01a	186988.670	2,2997	<0.001
**Doubling Time, t (days)**	2.96±0.00b	4.42±0.01c	-28.99±1.51a	468.989	2,2997	<0.001

Means ± SE within a row followed by different lowercase letters are significantly different at p = 0.05.

## Discussion

In this study, we aimed at measuring preference of the predatory mite *A*. *orientalis* between mites (*T*. *cinnabarinus*) and whiteflies (*B*. *tabaci*), and evaluating whether *A*. *orientalis* is able to establish its population when fed on whiteflies or on a mix of spider mites and whiteflies. Based on our results, *A*. *orientalis* randomly chose its prey between *T*. *cinnabarinus* and *B*. *tabaci*, although *B*. *tabaci* appeared to have a smaller nutritional value than *T*. *cinnabarinus*. Indeed *A*. *orientalis* population failed to increase when it fed on *B*. *tabaci*, but a prey mix with only 37.5% individuals of *T*. *cinnabarinus* resulted in *A*. *orientalis* producing approximately the same amount of offspring as when fed on *T*. *cinnabarinus* only.

Preferences of generalist predators are often tradeoffs between multiple factors, especially prey nutritional quality and the ease of attacking, this latter being influenced by a few factors, including the ease to detect and access the prey, prey abundance, and the defensive behavior of the prey, etc. [26, 39–40]. Some *Amblyseisus* mites prefer prey with higher nutritional levels. For example, Xu and Enkegaard (2010) reported higher prey preference of *A*. *swirskii* to 1^st^ instar larvae of western flower thrips, *Frankliniella occidentalis*, the prey with higher nutritional value [41], than to nymphs of two-spotted spider mite *Tetranychus urticae* [42]. Under natural conditions, phytoseiid mites often locate and choose prey patches based on host plant and prey specific herbivore-induced plant volatiles. These phytochemicals provide information to arthropod predators about prey quality [43]. However, within a small arena as the experimental unit used in the present study, volatiles induced by different prey species are mixed and might not impact prey selection. In addition, the small enclosed arena caused longer patch visits. Zhang and Sanderson (1993) investigated the searching behavior of three Phytoseiidae mite species, *Phytoseiulus persimilis*, *Metaseiulus* (= *Typhlodromus*) *occidentalis*, *Amblyseius andersoni*, and showed that the number of prey encountered and attacked per predator are positively correlated with the duration of patch visits [44]. They also suggested that starvation lead to longer patch visits of the predatory mites. In this case, it is possible that *A*. *orientalis* spent long time at each prey patch it randomly hit, and maximized its food intake regardless of the most abundant prey species in the patch. Some other generalist predators show similar foraging behaviors, such as *Macrolophus pygmaeus*. Previous studies show this predator has strong preference to the prey with higher relative density, regardless of its nutritional value [45–46].

The preference of *A*. *orientalis* between spider mites and whiteflies in our experiment may have been influenced by differences in the predator capacity to attack and handle these prey. Gan et al. (2001) reported that it took *A*. *orientalis* multiple attacks to kill a *P*. *citri* female adult, which lead to higher energy cost in preying on spider mites and possibly lead to a reduced preference [47]. However, spider mite protonymphs are much smaller in size than female adults and show much lower levels of defensive behaviors. *Amblyseius orientalis* also randomly chose its prey when eggs, larvae, and protonymphs of *T*. *cinnabarinus* were provided together, similar to the prey preferences of some other Phytoseiidae mites, such as *Neoseiulus californicus*, and *A*. *swirskii*, to different stages of *T*. *urticae* [48]. *Bemisia tabaci* eggs are of comparable sizes as *T*. *cinnabarinus* eggs, and both are smaller than *T*. *cinnabarinus* protonymphs. It will be interesting to further investigate prey preference of *A*. *orientalis* to the eggs of the two species when provided together. If a significant preference to *T*. *cinnabarinus* eggs is observed, the random selection between *T*. *cinnabarinus* nymphs and *B*. *tabaci* eggs might be attributed to a tradeoff between prey nutritional value and prey defensive behavior. Otherwise, random selection between the two types of eggs will suggest that prey nutritional value may not affect prey preference of *A*. *orientalis*.

Learning is another factor that might affect prey selection. Some predatory mite adults that experienced alternative food during their immature stages accept alternative food faster than naïve adults, eg. *P*. *persimilis* and *N*. *californicus* [49–50]. In our study, *A*. *orientalis* individuals used in the preference experiment were reared on *C*. *lactis* and naïve to *B*. *tabaci*, while *A*. *orientalis* individuals used in the *B*. *tabaci* and the prey mix treatments of the life table experiment experienced *B*. *tabaci* during their immature stages. If learning positively affects the predator capacity to recognize and handle prey, we would expect higher daily predation rates of *B*. *tabaci* in the life table experiment than in the preference experiments. However, *A*. *orientalis* female adults consumed 14.10 *B*. *tabaci* eggs per day in preference experiments, but only 8.05 *B*. *tabaci* eggs per day in the life table experiments. A possible reason is that adults in the preference experiments had been starved for 24 hours before being tested but not in the life table experiment. Predatory mites used in preference studies are often starved for a minimum of 24 hours to standardize their hunger level, with the impact of starvation on prey preference often considered as negligible [42, 51]. It is valuable to further investigate whether learning affects prey preference of *A*. *orientalis* or was confounded with the impact of starvation in the present study.

To further estimate the potential of *A*. *orientalis* in controlling whiteflies, we compared its development, longevity, fecundity, and intrinsic rate of increase with estimates of these parameters of *A*. *swirskii*, the commercially available biological control agent of *B*. *tabaci*, in Table 8. The intrinsic rate of increase of *A*. *orientalis* when fed on the prey mix is lower than that of *A*. *swirskii* on *B*. *tabaci*, but higher than that of *A*. *swirskii* on spider mites. An interesting result is that longevities, mainly owing to the post oviposition duration (Table 4), of *A*. *orientalis* that fed on *T*. *cinnabarinus* or on prey mix are much longer than either *A*. *orientalis* that fed on *B*. *tabaci*, or *A*. *swirskii*. Previous studies showed that longevity of predatory mite females are sometimes affected by their mating status. For example, Ji et al. (2007) indicated that *Neoseiulus cucumeris* that mated only once have longer post-oviposition durations and longevities than those that mated multiple times [52]. In addition, *A*. *orientalis* is also highly tolerant to starving. A previous study showed that it survived up to 16 days without food supply [53]. These facts suggested that *A*. *orientalis* might sustain its population for a long time in field trials with low prey density.

**Table 8:** Development, fecundity, and intrinsic rate of increase of *A*. *orientalis* and *A*. *swirskii* at 25±2°C.

Predator	Prey	Developmental Duration (days)*	Longevity	Proportion of Female Offspring	Daily Fecundity (eggs/female/day)	Cumulative Fecundity (eggs/female)	Intrinsic Rate of Increase (r_m_)	Data Source
*Amblyseius orientalis*								
	*T*. *cinnabarinus*		97.21	0.63	1.88	39.49	0.23	Present study
	*T*. *cinnabarinus + B*. *tabaci*	5.43	58.49	0.62	1.28	34.66	0.16	Present study
	*B*. *tabaci*	7.1	26.54	0.45	0.65	1.69	-0.03	Present study
*Amblseius swirskii*								
	*T*. *urticae*	6.47	23.82	-	1.18	13	0.13	Marzieh et al., 2014 [35]
	*B*. *tabaci*	8.3*	-	0.74	1	-	0.208	Nomikou et al., 2001 [13]
	*B*. *tabaci*	7.4*	-	0.64	1.66	-	0.213	Nomikou et al., 2002 [14]
	*Suidasia medanensis* (pontifica) (Acari: Suidasiidae)	5.01	22.14	0.65	1.71	22.52	0.222	Midthassel et al., 2013 [54]
	*C*. *lactis*	7	35.9	0.72	1.21	29.03	0.175	Nguyen et al., 2013 [55]
	*Cattail pollen*	7	25.8	0.69	-	16.1	0.14	Lee & Gillespie, 2011 [56]

*Developmental durations used in the table refer to the timing between egg laying and adult emergence (egg-adult), except for *A*. *swirskii* reared on *B*. *tabaci*, where it refers to egg-egg duration.

To compare the performance of the two predators when spider mites and whiteflies coexist, we also performed a preliminary experiment to estimate the predation of the two prey by *A*. *swirskii*, at similar combinations of prey densities as used in the preference study of *A*. *orientalis*. The methods and results of this experiment were summarized in supporting information (S1 Side Experiment). We found that when *A*. *swirskii* was reared on *B*. *tabaci* eggs, it consumed ca. 11.13 whiteflies during 12 hours, ca. 15% higher than that of *A*. *orientalis* (9.8). The mean preference index for *A*. *swirskii* to *B*. *tabaci* eggs did not differ significantly from that to *T*. *cinnabarinus* protonymphs (t = -0.669, df = 133, p = 0.504). A similar pattern was observed for *A*. *swirskii* consumption rate of each prey as affected by the prey proportion (Fig 2 and S1 Fig).

Overall, it is reasonable to consider *A*. *orientalis* as a potential biological control agent of *B*. *tabaci*, when whiteflies co-occur with *T*. *cinnabarinus*. For example, in vegetable greenhouses in Beijing, China and neighboring provinces (32–42°N and 110–120°E), *T*. *cinnabarinus* outbreaks often occur in spring (April to May), while *B*. *tabaci* outbreaks occur in early summer (June). Based on results of the present study, we believe it is valuable to evaluate strategies with releases of a specialist predator of *T*. *cinnabarinus*, such as *P*. *persimilis*, in combination or sequentially to releases of *A*. *orientalis*. The specialist predator is expected to control *T*. *cinnabarinus* before its outbreak, while *A*. *orientalis* is expected to establish its population before *T*. *cinnabarinus* is completely controlled, and to control *B*. *tabaci* in early summer. The ease of *A*. *orientalis* mass rearing allows this species to be mass released in greenhouses [Patent: CN 201110456703]. To achieve successful field applications, it will be necessary to further estimate possible impacts of abiotic and biotic factors on biological control efficiency of *B*. *tabaci*, including temperature, relative humidity, and interactions with other coexisting pests or predators.

## Supporting Information

S1 Dataset Fig 2 original data. (XLSX)

S2 Dataset Fig 3 original data. (XLSX)

S3 Dataset Table 1 original data. (XLSX)

S4 Dataset Table 2 original data (XLSX)

S5 Dataset Table 3 original data. (XLSX)

S6 Dataset Table 4 original data. (XLSX)

S7 Dataset Table 5 original data. (XLSX)

S8 Dataset Table 6 original data. (XLSX)

S9 Dataset Table 7 original data. (XLSX)

S10 Dataset Original data for S1 Fig. (XLSX)

S1 Fig Impact of the proportion of *T*. *cinnabarinus* and *B*. *tabaci* in the mixed diet provided to *A*. *swirskii*. (TIF)

S1 Side Experiment Preference of *A*. *swirskii* to *B*. *tabaci* eggs and *T*. *cinnabarinus* protonymphs. (DOCX)
