# Supplementary figures and images for: Prey Preference and Life Table of Amblyseius orientalis on Bemisia tabaci and Tetranychus cinnabarinus
Source: PLoS One. 2015 Oct 5;10(10):e0138820. doi: 10.1371/journal.pone.0138820 (PMC4593607; doi:10.1371/journal.pone.0138820)

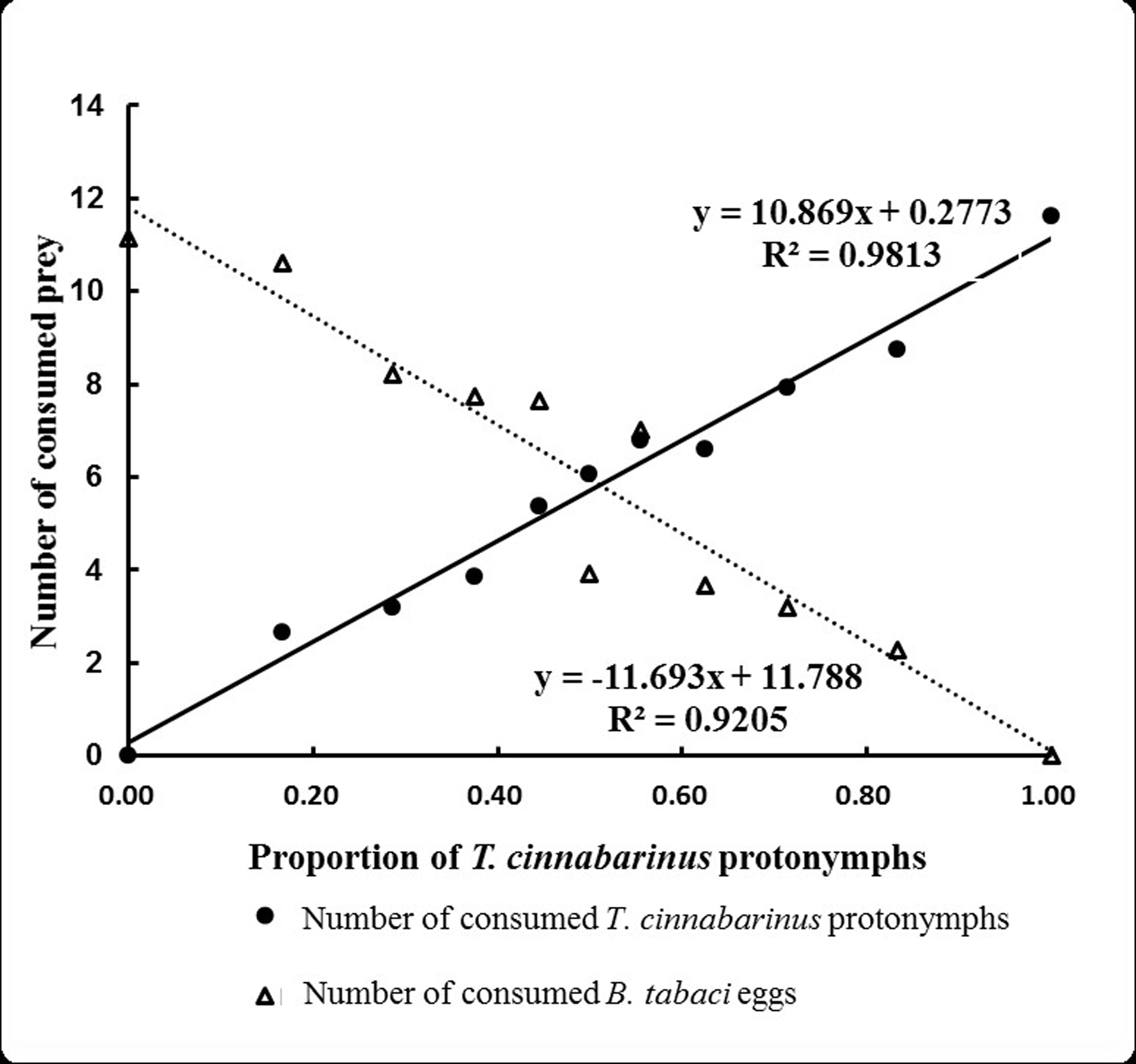

Supplement: S1 Fig — (TIF) [file pone.0138820.s011.tif]
