# Supplementary material for: Prey Preference and Life Table of Amblyseius orientalis on Bemisia tabaci and Tetranychus cinnabarinus
Source: PLoS One. 2015 Oct 5;10(10):e0138820. doi: 10.1371/journal.pone.0138820 (PMC4593607; doi:10.1371/journal.pone.0138820)
Supplement: S1 Side Experiment — (DOCX) [file pone.0138820.s012.docx]

**Preference of *A. swirskii* to *B. tabaci* eggs and *T. cinnabarinus* protonymphs**

**Materials and methods**

**Mites and whiteflies colonies**

*Amblyseius swirskii* (Athias-Henriot) (Acari: Phytoseiidae) was obtained from a commercial producer (Koppert Biological Systems, The Netherlands), and has been maintained on *Carpoglyphus lactis* (Linnaeus) (Acari: Carpoglyphidae) in a climate chamber at 25±1ºC, 80%±5% RH and 16L: 8D for multiple years. Other mites and pest species are the same as used in the main study.

**Prey preference** **between *B. tabaci* and *T. cinnabarinus***

The experiment was conducted to estimate the prey preference of *A. swirskii* between *B. tabaci* eggs and *T. cinnabarinus* protonymphs. Two sets of prey density combinations were provided to *A. swirskii* adult females starved for 24 hours: (1) 25 *B. tabaci* eggs and different numbers (5, 10, 15, 20, 25) of *T. cinnabarinus* protonymphs, and (2) 25 *T. cinnabarinus* protonymphs and different numbers (5, 10, 15, 20, 25) of *B. tabaci* eggs. A minimum of 14 replicates were prepared for each density combination. The number of each prey species consumed within 12 hours was recorded.

The prey preference index (α) was estimated using the same method as used in the main study (Eq. 1). Linear regressions were conducted to estimate the impact of the proportion of *T. cinnabarinus* in the prey mix on the number of both prey species consumed by *A. swirskii*. All analyses were processed with SPSS 19.0.

**Results**

**Prey preference between *B. tabaci* and *T. cinnabarinus***

When *B. tabaci* eggs and *T. cinnabarinus* protonymphs were provided together, The mean preference index for *A. swirskii* to *B. tabaci* eggs (α ±SE:0.76±0.03) did not differ significantly from that to *T. cinnabarinus* protonymphs (α ±SE:0.79±0.03) (t=-0.669, df =133, p=0.504). The number of each prey type consumed matched their available proportion (S2 Figure). The results suggest that *A. swirskii* randomly preys on *B. tabaci* eggs and *T. cinnabarinus* protonymphs.

**S1 Fig.** **Impact of the proportion of *T. cinnabarinnus* and *B. tabaci* in the mixed diet provided to *A. swirskii*.**
